# Integration of Sequence Data from a Consanguineous Family with Genetic Data from an Outbred Population Identifies *PLB1* as a Candidate Rheumatoid Arthritis Risk Gene

**DOI:** 10.1371/journal.pone.0087645

**Published:** 2014-02-10

**Authors:** Yukinori Okada, Dorothee Diogo, Jeffrey D. Greenberg, Faten Mouassess, Walid A. L. Achkar, Robert S. Fulton, Joshua C. Denny, Namrata Gupta, Daniel Mirel, Stacy Gabriel, Gang Li, Joel M. Kremer, Dimitrios A. Pappas, Robert J. Carroll, Anne E. Eyler, Gosia Trynka, Eli A. Stahl, Jing Cui, Richa Saxena, Marieke J. H. Coenen, Henk-Jan Guchelaar, Tom W. J. Huizinga, Philippe Dieudé, Xavier Mariette, Anne Barton, Helena Canhão, João E. Fonseca, Niek de Vries, Paul P. Tak, Larry W. Moreland, S. Louis Bridges, Corinne Miceli-Richard, Hyon K. Choi, Yoichiro Kamatani, Pilar Galan, Mark Lathrop, Towfique Raj, Philip L. De Jager, Soumya Raychaudhuri, Jane Worthington, Leonid Padyukov, Lars Klareskog, Katherine A. Siminovitch, Peter K. Gregersen, Elaine R. Mardis, Thurayya Arayssi, Layla A. Kazkaz, Robert M. Plenge

**Affiliations:** 1 Division of Rheumatology, Immunology, and Allergy, Brigham and Women's Hospital, Harvard Medical School, Boston, Massachusetts, United States of America; 2 Division of Genetics, Brigham and Women's Hospital, Harvard Medical School, Boston, Massachusetts, United States of America; 3 Program in Medical and Population Genetics, Broad Institute, Cambridge, Massachusetts, United States of America; 4 Department of Human Genetics and Disease Diversity, Tokyo Medical and Dental University Graduate School of Medical and Dental Sciences, Tokyo, Japan; 5 Laboratory for Statistical Analysis, Center for Integrative Medical Sciences, RIKEN, Yokohama, Japan; 6 New York University Hospital for Joint Diseases, New York, New York, United States of America; 7 Molecular Biology and Biotechnology Department, Human Genetics Division, Damascus, Syria; 8 The Genome Institute, Washington University School of Medicine, St. Louis, Missouri, United States of America; 9 Department of Biomedical Informatics, Vanderbilt University School of Medicine, Nashville, Tennessee, United States of America; 10 Department of Medicine, Albany Medical Center and The Center for Rheumatology, Albany, New York, United States of America; 11 Division of Rheumatology, Department of Medicine, New York, Presbyterian Hospital, College of Physicians and Surgeons, Columbia University, New York, New York, United States of America; 12 Department of Medicine, Vanderbilt University School of Medicine, Nashville, Tennessee, United States of America; 13 The Department of Psychiatry at Mount Sinai School of Medicine, New York, New York, United States of America; 14 Center for Human Genetics Research, Massachusetts General Hospital, Harvard Medical School, Boston, Massachusetts, United States of America; 15 Department of Human Genetics, Radboud University Medical Centre, Nijmegen, The Netherlands; 16 Department of Clinical Pharmacy and Toxicology, Leiden University Medical Center, Leiden, The Netherlands; 17 Department of Rheumatology, Leiden University Medical Centre, Leiden, The Netherlands; 18 Service de Rhumatologie et INSERM U699 Hôpital Bichat Claude Bernard, Assistance Publique des Hôpitaux de Paris, Paris, France; 19 Université Paris 7-Diderot, Paris, France; 20 Institut National de la Santé et de la Recherche Médicale (INSERM) U1012, Université Paris-Sud, Rhumatologie, Hôpitaux Universitaires Paris-Sud, Assistance Publique-Hôpitaux de Paris (AP-HP), Le Kremlin Bicêtre, France; 21 Arthritis Research UK Epidemiology Unit, Centre for Musculoskeletal Research, University of Manchester, Manchester Academic Health Science Centre, Manchester, United Kingdom; 22 Rheumatology Research Unit, Instituto de Medicina Molecular, Faculdade de Medicina da Universidade de Lisboa, Lisbon, Portugal; 23 Rheumatology Department, Santa Maria Hospital–CHLN, Lisbon, Portugal; 24 Department of Clinical Immunology and Rheumatology & Department of Genome Analysis, Academic Medical Center/University of Amsterdam, Amsterdam, The Netherlands; 25 Department of Clinical Immunology and Rheumatology, Academic Medical Center/University of Amsterdam, Amsterdam, The Netherlands; 26 GlaxoSmithKline, Stevenage, United Kingdom; 27 Division of Rheumatology and Clinical Immunology, University of Pittsburgh, Pittsburgh, Pennsylvania, United States of America; 28 Division of Clinical Immunology and Rheumatology, Department of Medicine, University of Alabama at Birmingham, Birmingham, Alabama, United States of America; 29 Channing Laboratory, Department of Medicine, Brigham and Women's Hospital, Harvard Medical School, Boston, Massachusetts, United States of America; 30 Section of Rheumatology, Boston University School of Medicine, Boston, Massachusetts, United States of America; 31 Clinical Epidemiology Research and Training Unit, Boston University School of Medicine, Boston, Massachusetts, United States of America; 32 Centre d'Etude du Polymorphisme Humain (CEPH), Paris, France; 33 Université Paris 13 Sorbonne Paris Cité, UREN (Nutritional Epidemiology Research Unit), Inserm (U557), Inra (U1125), Cnam, Bobigny, France; 34 McGill University and Génome Québec Innovation Centre, Montréal, Canada; 35 Program in Translational NeuroPsychiatric Genomics, Institute for the Neurosciences, Department of Neurology, Brigham and Women's Hospital, Boston, Massachusetts, United States of America; 36 NIHR Manchester Musculoskeletal Biomedical, Research Unit, Central Manchester NHS Foundation Trust, Manchester Academic Health Sciences Centre, Manchester, United Kingdom; 37 National Institute for Health Research, Manchester Musculoskeletal Biomedical Research Unit, Central Manchester University Hospitals National Health Service Foundation Trust, Manchester Academic Health Sciences Centre, Manchester, United Kingdom; 38 Rheumatology Unit, Department of Medicine (Solna), Karolinska Institutet, Stockholm, Sweden; 39 Lunenfeld-Tanenbaum Research Institute, Mount Sinai Hospital, Toronto, Canada; 40 Toronto General Research Institute, Toronto, Canada; 41 Department of Medicine, University of Toronto, Toronto, Canada; 42 The Feinstein Institute for Medical Research, North Shore–Long Island Jewish Health System, Manhasset, New York, United States of America; 43 Weill Cornell Medical College-Qatar, Education City, Doha, Qatar; 44 Tishreen Hospital, Damascus, Syria; 45 Syrian Association for Rheumatology, Damascus, Syria; University of Michigan, United States of America

## Abstract

Integrating genetic data from families with highly penetrant forms of disease together with genetic data from outbred populations represents a promising strategy to uncover the complete frequency spectrum of risk alleles for complex traits such as rheumatoid arthritis (RA). Here, we demonstrate that rare, low-frequency and common alleles at one gene locus, *phospholipase B1* (*PLB1*), might contribute to risk of RA in a 4-generation consanguineous pedigree (Middle Eastern ancestry) and also in unrelated individuals from the general population (European ancestry). Through identity-by-descent (IBD) mapping and whole-exome sequencing, we identified a non-synonymous c.2263G>C (p.G755R) mutation at the *PLB1* gene on 2q23, which significantly co-segregated with RA in family members with a dominant mode of inheritance (*P* = 0.009). We further evaluated *PLB1* variants and risk of RA using a GWAS meta-analysis of 8,875 RA cases and 29,367 controls of European ancestry. We identified significant contributions of two independent non-coding variants near *PLB1* with risk of RA (rs116018341 [MAF = 0.042] and rs116541814 [MAF = 0.021], combined *P* = 3.2×10^−6^). Finally, we performed deep exon sequencing of *PLB1* in 1,088 RA cases and 1,088 controls (European ancestry), and identified suggestive dispersion of rare protein-coding variant frequencies between cases and controls (*P* = 0.049 for C-alpha test and *P* = 0.055 for SKAT). Together, these data suggest that *PLB1* is a candidate risk gene for RA. Future studies to characterize the full spectrum of genetic risk in the *PLB1* genetic locus are warranted.

## Introduction

Rheumatoid arthritis (RA [MIM 180300]) is a chronic autoimmune disease with destruction of synovial joints affecting up to 1% of the population worldwide [1]. RA is a heritable disease, and recent genome-wide association studies (GWAS) and related approaches have identified more than 50 RA susceptibility loci [2]–[4]. However, as is true for most complex traits, a substantial proportion of genetic heritability of RA remains unexplained [5], [6]. Simulated data based on GWAS [6] and empirical data from direct sequencing [7] indicate that a mixture of common, low-frequency and rare variants contribute to risk of RA. Indeed, there are now examples of several gene loci that harbor multiple RA risk alleles (from common to rare) [7]. These data suggest that other gene loci also contain multiple RA risk alleles, and integrating deep-sequencing data with dense genotyping data in large patient collections may be a useful strategy to uncover new RA risk loci.

Whole-exome sequencing and identity-by-descent (IBD) mapping of ancestral haplotypes is emerging as a powerful approach to identify rare causal mutations in families with highly penetrant forms of disease [8], [9]. In some instances, the same genes that harbor rare mutations that cause disease in families also harbor other risk variants that influence risk of the same or related diseases in outbred populations [10]. Jordan *et al*. identified causal mutations in *CARD14* through the exome sequencing study of a pedigree affected with a Mendelian form of psoriasis [11], and they also reported both rare and common risk variants of *CARD14* in the general psoriasis case-control cohort [12]. Furthermore, a recent GWAS meta-analysis validated *CARD14* as a psoriasis risk locus [13]. Al-Mayouf *et al.* identified a loss-of-function variant in *DNASE1L3* responsible for a familial form of systemic lupus erythematosus (SLE) [14]. *DNASE1L3* was also identified as a gene in an SLE risk locus from GWAS [15]. Contributions of the variants in the maturity-onset diabetes of the young (MODY) genes on type 2 diabetes susceptibility, or the familial hypercholesterolemia (FH) genes on cardiovascular diseases are also well known [16], [17].

There are few previous reports of highly penetrant forms of familial RA [18]. Here, we report a consanguineous pedigree with a Mendelian form of RA. Through the integration of IBD mapping and whole-exome sequencing, we identify a rare mutation associated with risk of RA in the *phospholipase B1* (*PLB1*) gene at chromosome 2p23. Our study also demonstrates significant contributions of coding and non-coding variants in *PLB1* on the risk of RA in an outbred population of European ancestry.

## Results

### The consanguineous pedigree with a Mendelian form of RA

**Figure 1:**
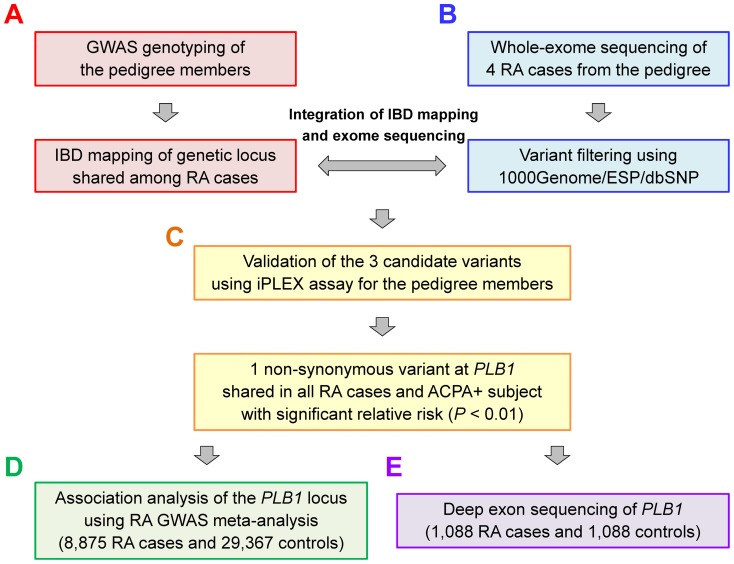
Description of the study design. Our study consists of analysis on three sources of data: (1) rare risk variant detection in the consanguineous pedigree with Mendelian form of RA (A–C), (2) regional association analysis using RA GWAS meta-analysis of European populations (D), and (3) target deep exon sequencing of the European RA case-control cohort (E). (A) We conducted IBD mapping of the pedigree using genome-wide SNP genotype data. (B) Whole-exome sequencing was performed for the 4 affected RA cases of the pedigree. (C) By integrating the results of IBD mapping and whole-exome sequencing, and subsequently conducting the validation assay, we identified a non-synonymous mutation of *PLB1* associated with RA segregation. (D) We evaluated the regional association of the *PLB1* locus using RA GWAS meta-analysis including 8,875 RA cases and 29,367 controls. (E) Deep exon sequencing of *PLB1* and gene-based rare variant test was conducted for 1,088 RA cases and 1,088 controls.

**Figure 2:**
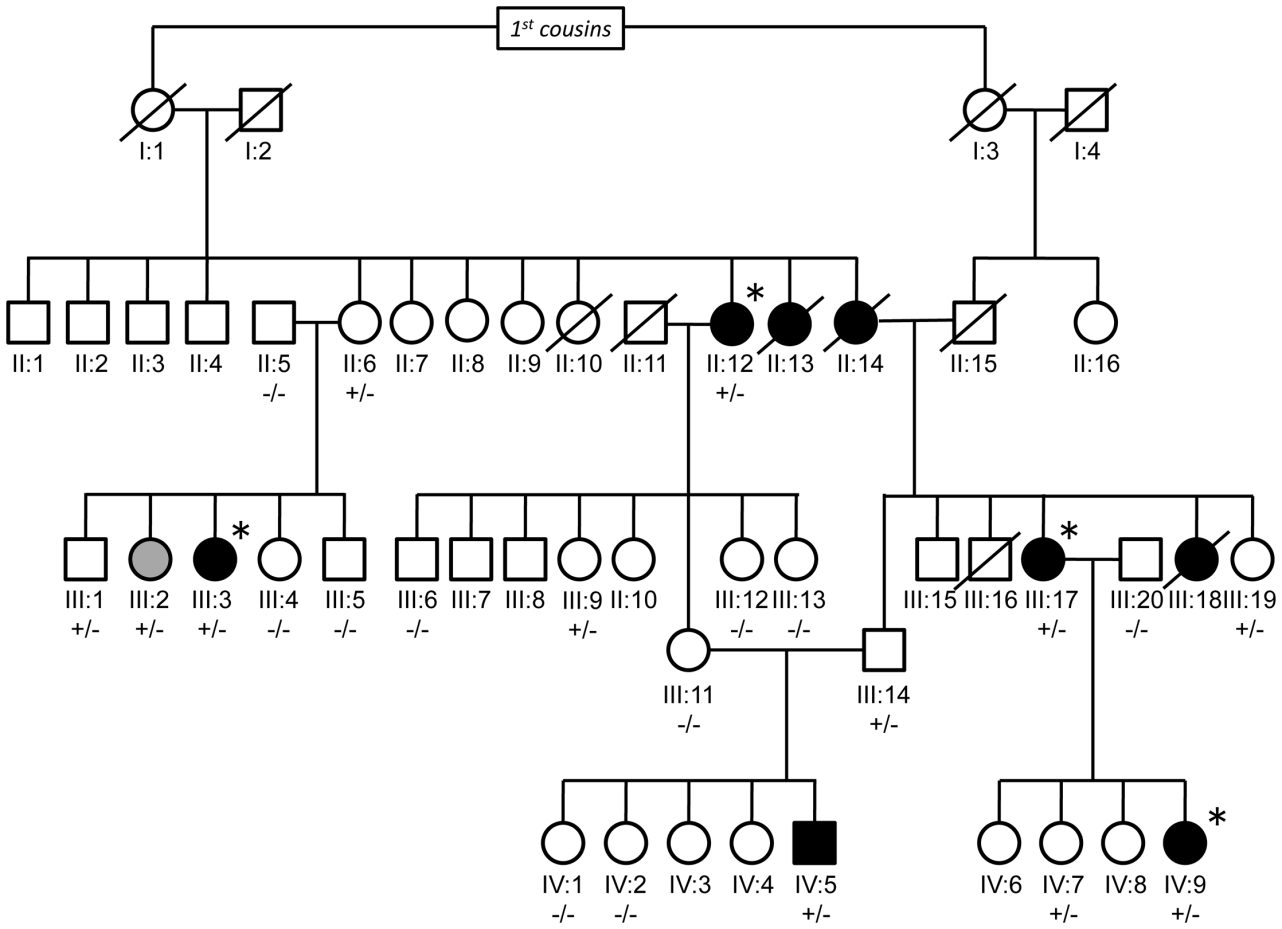
The consanguineous pedigree with Mendelian form of RA. The consanguineous pedigree consists of 49 individuals from 4 generations. The pedigree included 8 individuals affected with RA (colored in black) and 1 ACPA-positive unaffected subject subject (colored in gray). Four RA cases for whom whole-exome sequencing was conducted were indicated with asterisks. Genotypes of the identified *PLB1* p.G755R mutation was indicated by the combination of “+” (mutated allele) and “-” (reference allele).

We report a newly identified, 4-generation, consanguineous pedigree from the Middle East in which 8 of 49 individuals are affected with RA (Figure 1, Figure 2, and Table S1). No other family members were found. Each affected case has symmetrical polyarthritis and is positive for anti-citrullinated protein antibodies (ACPA), a key component of the RA classification criteria related to higher disease severity [19]. The proportion of affected cases in the pedigree members (16.3%) was much higher than the population prevalence of RA in the Middle East (∼1%). The average age of onset (35.4 years) was also younger compared to those observed in the general European or Middle East populations [1], [20]. Together, these observations suggested contribution of pedigree-specific risk factors of RA not explained by known genetic or environmental factors, including *HLA-DRB1* shared epitope (SE) alleles (odds ratio [OR]  = 2.47 in the Syrian population [20]). Among the living unaffected subjects of the pedigree, one subject was strongly positive for ACPA (≥60 units; Table S1). Considering the high specificity of ACPA to RA (>95%) and that ACPA can be found in RA case sera over 10 years prior to the diagnosis of the disease [19], [21], we classified this one ACPA-positive unaffected subject into the “affected” case group. (We note that results from the IBD mapping and the validation genotyping did not change substantially when we excluded this ACPA-positive unaffected subject from the analysis.)

### Whole-genome SNP genotyping and IBD mapping

We anchored our overall study design (Figure 1) based on a model in which a rare mutation was responsible for RA risk in the consanguineous family. Based on the segregation of RA (Figure 2), we initially posited an autosomal recessive mode of inheritance (Figure 1A). Among the 24 family members available to study (5 ACPA-positive RA cases, 1 ACPA-positive unaffected subject, and 18 ACPA-negative unaffected subjects), we used GWAS data to conduct homozygosity mapping. However, we did not observe any IBD regions shared among all 5 ACPA-positive RA cases and 1 ACPA-positive unaffected subject. This result suggested that a different genetic model (e.g., autosomal dominant mode with incomplete penetrance) may be responsible for RA risk in this family.

**Figure 3:**
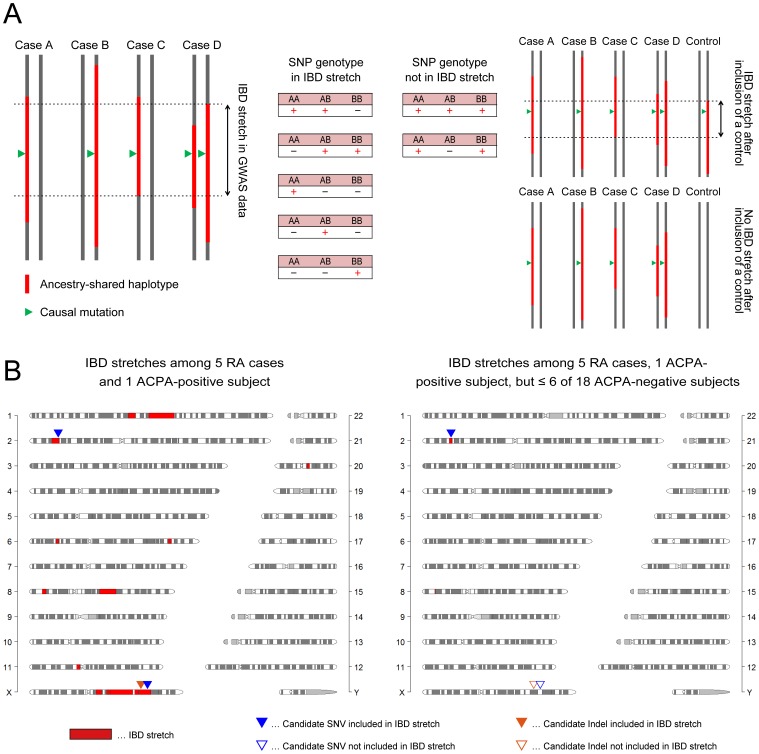
IBD mapping of the pedigree with RA. (A) We investigated the novel non-parametric linkage analysis method which enabled the IBD mapping for the disease with any types of inheritance modes. Affected cases should carry one or two copy of the mutation which resides on a single ancestral haplotype in IBD, thus, SNPs adjacent to the causal mutation lose homozygous genotypes for at least one of the alleles. Our method searched the regional IBD stretches where SNP genotypes of the affected cases followed this rule, and then imputed presence or absence of the ancestral haplotype in the other unaffected subjects separately. (B) Results of the IBD mapping in the consanguineous pedigree with RA. Mapped IBD stretches are indicated as the bands colored in red. As the pedigree members used for the IBD mapping increased (left panel; 5 RA cases and 1 ACPA-positive unaffected subject, right panel; all available subjects), IBD stretches narrowed down (see detailed process in Table S2). Candidate causal SNVs and Indels obtained after whole-genome exome sequencing were indicated as the triangles colored in blue and orange, respectively. The variants included and not included in the IBD stretches of each stages are indicated with filled and non-filled colors. Finally, only one SNV at 2p23 was included in the defined IBD stretch (right panel).

To search for a genetic mutation that may confer risk of RA under a different mode of inheritance, we developed and applied a newly investigated non-parametric linkage analysis (Figure 3A). This method, which is based on the “SNP streak” approach to assess homogeneity of the adjacent SNP genotypes on the ancestral haplotype [22], [23], is applicable to any type of inheritance mode without prior estimation of mutation penetrance. We further extended the method to utilize genotype information from unaffected pedigree members as well as affected cases. The principle of our method is that affected cases should carry at least one copy of the mutation which resides on a single ancestral haplotype in IBD, but never homozygous for the non-mutated allele [22], [23]. Therefore, genetic markers adjacent to the causal mutation lose homozygous genotypes for at least one of the alleles (Figure 3A). We used genome-wide SNP data to search the regional IBD stretches that lose one or both homozygous genotypes in affected cases, which serve as candidate regions harboring causal rare mutations. Our method identified 14 IBD stretches spanning 115.9 Mbp (3.7% of human genome) shared in at least one copy of the haplotype among 5 RA cases and 1 ACPA-positive unaffected subject (Figure 3B left panel and Table S2).

To further narrow the number of critical regions as well as the size of each region, we applied our IBD mapping method to use GWAS data in ACPA-negative unaffected subjects to test the presence of each of the 14 IBD stretches. We hypothesized that the IBD region containing a causal mutation in this family would be shared by a smaller number of the ACPA-negative unaffected subjects compared to the other IBD regions not containing the mutation. We confined the IBD regions by consecutively restricting the number of the ACPA-negative unaffected subjects harboring the IBD region (see detailed process in Table S2). By this approach, we narrowed the mapped IBD regions into a single stretch with 2.4 Mb length (0.08% of the genome) at 2p23 (from 27.2–29.5 Mb), which was shared among 5 RA cases and 1 ACPA-positive unaffected subject but ≤6 of 18 ACPA-negative unaffected subjects (Figure 3B right panel). We note that the probability to observe at least one IBD region in ≤6 of 18 unaffected subjects was not significant in this pedigree (permutation *P* = 0.38).

### Variant filtering in the whole-exome sequencing and overlap with the IBD stretches

To identify the causal mutation in the 2.4 Mb region at 2p23 (which contains 56 protein-coding genes), we performed whole exome sequencing in 4 of the ACPA-positive RA cases (Figure 1B). We did not sequence the rest of one ACPA-positive RA case, since genomic DNA of this subject was not available at the time of sequencing. After whole-exome sequencing, read alignment, and variant calling, we isolated 65,524 variants genome-wide, with average depth of ×290.1 and Ti/Tv ratio of 2.74. On average, 94.0% of the targeted regions (∼45 Mbp) yielded ≥10X coverage. Genotype concordance rates of the identified variants commonly included in the whole-genome SNP genotyping results were as high as 99.56% (range = 99.47% to 99.61% for each sample). Within the 2.4 Mb critical region on 2p23, we identified 168 variants with 99.4% coverage of coding exons at ≥10X coverage. We did not find any copy number variant (CNV) shared among RA cases in this region.

We applied stringent filtering criteria to select for rare pathogenic variants present in this family but not in any public database with non-reference allele frequency ≥0.01 (dbSNP v132, 1000 Genomes Project Phase I data [24], and NHLBI Grand Opportunity Exome Sequencing Project [ESP] 5400 [25]). Of the 168 protein-coding variants within the critical region on 2p23, only one variant was identified by our filtering approach, a missense mutation in the *phospholipase B1* (*PLB1*) gene (Figure 3B right panel). For completeness, we also evaluated the other 13 IBD stretches shared in at least one copy among all 5 RA cases and 1 ACPA-positive unaffected subject, and found one additional missense single-nucleotide variant (SNV) and 1 insertion-deletion (Indel) included in the IBD stretches (Table 1). While we considered all 3 variants to be possibly causal in this family, we considered the one missense mutation in the IBD stretch at 2p23 to be the most promising candidate causal mutation. A full list of the filtered variants from whole exome sequencing is provided in Table S3.

**Table 1:** Results of the validation assay for candidate variants derived from exome sequencing.

			Allele		Possession of the variant in family members		
Gene^a^	Chr	Position (bp)^b^	Ref/Alt	Amino acid change	5 RA cases and 1 ACPA+ unaffected subject	16 ACPA- unaffected subjects	*P* c
*PLB1*	2	28,816,563	G/C	G755R	6/6	6/16	0.0090
*ANKRD58*	X	118,893,513	G/A	G295S	6/6	9/16	0.087
*AMOT*	X	112,022,297	C/CAGG	P1028PL	6/6	14/16	0.74

a Genes of which variants were shared among 5 RA cases and 1 ACPA+ unaffected subject are indicated.

b Based on NCBI Build 37/hg19.

c Mid-P value of Fisher's exact test for RA cases and unaffected subjects are indicated.

RA; rheumatoid arthritis, ACPA; anti-citrullinated protein antibodies.

### RA risk mutation of *PLB1* in the consanguineous pedigree

To technically confirm the variants identified by whole exome-sequencing, and also to evaluate the segregation pattern of the variants in this family (with an emphasis on the 2p23 variant), we genotyped each of the 3 candidate variants in all available 22 family members (Figure 1C). As expected based on the initial GWAS data and IBD mapping, we found that only the *PLB1* missense mutation co-segregated with RA in the pedigree without Mendelian error. The mutation was observed in all the 5 ACPA-positive RA cases and 1 ACPA-positive unaffected subject while only 6 of 16 ACPA-negative unaffected subjects inherited the *PLB1* mutation (*P* = 0.009; Figure 2 and Table 1). None of the family members was homozygous for this mutation. These observations are consistent with a dominant inheritance mode with a penetrance of 0.50.

**Figure 4:**
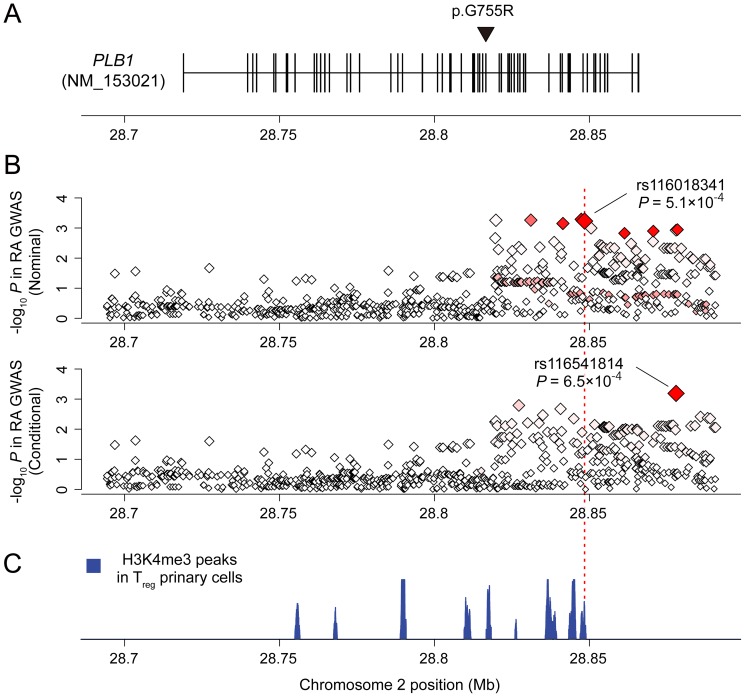
Association of the *PLB1* locus in RA GWAS meta-analysis. (A) Coding regions of *PLB1* and p.G755R mutation identified in the consanguineous RA pedigree. *PLB1* consists of 58 exons (NM_153021), and p.G755R (c.2263G>C) mutation was located at exon 33 (the black triangle). (B) Regional association of *PLB1* in RA GWAS meta-analysis including 8,875 RA cases and 29,367 controls from the European populations. Upper panel showed the results of nominal association, and the lower panel showed the results of conditional analysis with rs116018341, the top SNP in the nominal associations. The red diamond-shaped dots represent P-values of the SNPs in the GWAS meta-analysis, and the intensity of the red color in the dots represents the *r^2^* value with the most significantly associated SNP. Stepwise logistic regression analysis demonstrated multiple independent signals driven by non-coding variants. (C) H3K4me3 peak of T_reg_ primary cells in the *PLB1* locus. Non-coding RA risk SNP of rs116018341 overlapped with one of the H3K4me3 peaks as the SNP located in the most vicinity of the peak summit (a vertical dashed red line).

*PLB1* consists of 58 exons (NM_153021), and the mutation was identified at exon 33 (c.2263G>C [p.G755R]; Figure 4). This mutation was highly conserved (GERP score [26]  = 4.02), and predicted to be “tolerated” by SIFT [27] but “probably damaging” by PolyPhen-2.0 [28]. This mutation was not registered at any of the databases (dbSNP v132, 1000 Genomes Phase I [24], or ESP5400 [25]), and not present in the result of deep exon sequencing of *PLB1* in the European RA case-control cohort (see below).

### GWAS meta-analysis of the *PLB1* locus and RA risk of non-coding variants

To provide additional support for the role of *PLB1* in risk of RA, we evaluated common (minor allele frequency [MAF] >5%) and low-frequency variants (MAF 1–5%) near the *PLB1* gene locus in an outbred population of European ancestry (Figure 1D). We used a GWAS of 8,875 seropositive RA cases and 29,367 controls from 11 studies (Table S4). Five of the GWAS datasets were previously unpublished (3,427 cases and 6,837 controls from ReAct, Dutch, anti-TNF response to therapy collection [ACR-REF], CORRONA, and Vanderbilt) [29]–[33], which increased the power of our published dataset (Figure 4 and Table 2) [2], [4], [7]. We observed no evidence of systematic bias genome-wide (inflation factor λ_GC_ = 1.034; Table S4). We applied genotype imputation using the 1000 Genome Project reference panel [24], and assessed the *PLB1* locus with a set of densely fine-mapped SNPs (919 SNPs for ±50 kbp of *PLB1*; average SNP interval = 0.27 kbp). We considered a value of *P*<9.0×10^−4^ as statistically significant based on permutation analysis of all SNPs in the region.

**Table 2:** Results of the GWAS meta-analysis of European RA case-control cohorts in the *PLB1* locus.

				A1 Freq.			
rsID	Chr	Position (bp)^a^	A1/A2	RA cases (*n* = 8,875)	Controls (*n* = 29,367)	OR (95%CI)	*P*
rs116018341	2	28,848,761	A/C	0.045	0.041	1.18 (1.07–1.29)	5.1×10^−4^
rs116541814^b^	2	28,877,974	A/G	0.022	0.020	1.34 (1.13–1.59)	6.5×10^−4^
rs116018341-rs116541814 haplotype^c^	2	-	AG or CA/CG	0.067	0.062	1.21 (1.12–1.32)	3.2×10^−6^

a Based on NCBI Build 37/hg19.

b Conditioned with rs116018341.

c AA risk haplotype was not observed in the imputation reference panel.

RA; rheumatoid arthritis, ACPA; anti-citrullinated protein antibodies.

The most strongly-associated signal was observed at a low-frequency intronic SNP in *PLB1*, rs116018341 (MAF = 0.041, OR = 1.18, *P* = 5.1×10^−4^; Figure 4B upper panel), which surpassed our permutation-based threshold of significance. To determine if additional variants also contributed to risk of RA, we performed forward-type step-wise logistic regression analysis. We found evidence for a second, independent association of a low-frequency *PLB1* intronic SNP, rs116541814 (MAF = 0.020, OR = 1.34, *P* = 6.5×10^−4^; Figure 4B lower panel). After conditioning on these two variants, no significant regional association was observed (*P*>0.01 for all remaining SNPs).

We built haplotypes containing these two SNPs to test a combined genetic model. The common haplotype including either of the risk alleles for these 2 SNPs demonstrated a significant association with RA risk (MAF = 0.062, OR = 1.21, *P* = 3.2×10^−6^). These two intronic SNPs were not in linkage disequilibrium (LD) with any of the protein-coding variants of *PLB1* (*r^2^*<0.3 for common variants [MAF ≥0.05] and *r^2^*<0.1 for low-frequency or rare variants [MAF<0.05]), suggesting that observed RA risk was primarily derived from non-coding variants of *PLB1*.

In order to assess the functional contribution of the non-coding variants, we evaluated overlap of the RA risk SNPs with trimethylation of histone H3 at lysine 4 (H3K4me3) peak of primary CD4^+^ regulatory T cells (T_reg_ cells). The H3K4me3 mark is particularly informative for cell-type specific overlap with trait-associated variants, and RA risk variants showed significant enrichment in T_reg_ primary cells [34]. The RA risk SNP of rs116018341 (and the SNPs in absolute LD with it; *r^2^* = 1.00), was within one of the H3K4me3 peaks of T_reg_ primary cells (*P* = 0.043), while the other risk SNP, rs116541814, was not (Figure 4C). In a search of public eQTL databases (eQTL Browser and Blood eQTL Browser [35], see URL), we found no evidence that either SNP (or SNPs in LD with them, *r^2^*>0.8) influenced *PLB1* gene expression.

### Deep exon sequencing of *PLB1* and RA risk of protein-coding variants

Finally, we sequenced the coding exons of *PLB1* to search for independent rare variants that may contribute to risk of RA in an outbred population of European ancestry (Figure 1E). Deep exon sequencing was performed in 1,088 RA cases and 1,088 genetically-matched controls from the European populations, as a part of the Pharmacogenomics Research Network (PGRN) sequencing project [Diogo D. et al. Manuscript in preparation]. Overall, 96% of the targeted regions were sequenced with ≥20X coverage. We obtained 102 coding variants (i.e. variants annotated as synonymous, missense, or nonsense) in *PLB1*, of which 92 had MAF<0.01 in controls.

To test for significance, we applied gene-based tests (the burden test, variable threshold test [36], frequency-weighted test [37], C-alpha test [38], and sequence kernel association test [SKAT] [39]) for all rare coding variants with MAF<0.01 (Table 3 and Table S5). We observed suggestive enrichment of rare variants in the protein-coding region of *PLB1* (*P* = 0.049 for C-alpha test [38], and *P* = 0.055 for SKAT [39]). Both of these gene-based tests allow for opposite directional effects of the variants (two-sided test). In contrast, the gene-based tests which assume same directionality of effects of the variants (one-sided test) did not show significant results (*P*>0.30 for the burden test, variable threshold test [36], and frequency-weighted test [37]). Association signals in two-sided tests were more apparent for synonymous variants (*n* = 30, *P*<0.022), but not significant for non-synonymous variants (*n* = 62, *P*>0.30).

**Table 3:** Results of rare variant tests for *PLB1* coding variants in the European RA case-control cohort.

	Association analysis				
	One-sided test			Two-sided test	
No. variants^a^	BURDEN	VT	FRQWGT	CALPHA	SKAT
92	0.33	0.64	0.60	0.049	0.055

a Low-frequency rare coding variant (MAF≤0.01) obtained from deep sequencing of 1,088 RA cases and 1,088 controls were selected.

RA; rheumatoid arthritis, ACPA; anti-citrullinated protein antibodies.

BURDEN; burden test, VT; variable threshold test, FRQWGT; frequency-weighted test, CALPHA; C-alpha test, SKAT; sequence kernel association test.

## Discussion

Three lines of evidence suggest that coding and non-coding alleles at *PLB1* contribute to risk of RA. First, IBD mapping and whole-exome sequencing of a consanguineous Mendelian pedigree from the Middle East identified a rare non-synonymous mutation in *PLB1* (p.G755R). The mutation co-segregated with RA in dominant inheritance with incomplete penetrance but significant relative risk (*P* = 0.009). Second, large-scale RA GWAS meta-analysis in Europeans identified two independent non-coding variants near *PLB1*, which constitute a common risk haplotype associated with risk of RA (*P* = 3.2×10^−6^). Third, targeted exon sequencing of *PLB1* in Europeans demonstrated suggestive association of rare coding variants with risk of RA (*P*<0.05). Together, contributions of rare, low-frequency, and common alleles of *PLB1* observed in inbred and outbred populations of different ancestry provide supportive evidence that *PLB1* is a RA risk gene.

Identification of rare causal variants that contribute to complex disease etiology is an important issue in human genetics. Given the expected effect size of rare variants, extremely large sample sizes are required to identify disease-associated rare variants in studies of complex traits in outbred populations alone [40]. Alternatively, assessment of rare causal mutations in pedigrees with Mendelian forms of common disease, and validation of the identified gene in the outbred patient populations, could be an efficient approach. Our study provides support for this approach in RA, thereby complementing findings from previous studies in other diseases [10]–[17].

PLB1 is an enzyme that has both phospholipase A1 and A2 enzymatic activities. The PLB1 protein contains 3 GDSL-like lipase (acylhydrolase) domains (Figure S1) [41], [42]. The p.G755R mutation identified in the consanguineous RA pedigree was located within the second GDSL-like lipase domain (amino acid positions 741 to 1015). GDSL-like lipase domain has essential biological roles of PLB1 protein as lysophopholipase [42], and localizations of the RA risk variant on it might imply their functional impact on the enzymatic activity of PLB1.

The functional etiology of *PLB1* in human disease pathogenesis has not been well investigated. There exist a few reports suggesting contribution of *PLB1* and other phospholipase family genes on human autoimmune disease. The *PLB1* locus has suggestive evidence as a type 1 diabetes risk locus (*P*<10^−6^ for the SNP located 70 kbp upstream of *PLB1*) [43]. Duan *et al.* reported that expression of *PLB1* is upregulated in peripheral blood mononuclear cells (PBMCs) of patients with ankylosing spondylitis (an autoimmune disease that shares clinical features with RA) compared to healthy controls [44]. Recently, whole-exome sequencing analysis on a pedigree with a dominantly inherited immunodeficiency and autoimmunity identified a causal mutation in a gene related to *PLB1*, the phospholipase Cγ2 (*PLCG2*) gene [45]. Further studies assessing functional impacts of the *PLB1* mutations on RA pathogenesis are required.

Beyond the novel finding of *PLB1* as a candidate RA risk gene, our study developed and applied novel statistical methodologies. We developed a non-parametric linkage analysis method that enables IBD mapping in a pedigree with any mode of inheritance. Our method utilized genotype information of both affected and unaffected subjects without requiring prior estimation of penetrance. Due to its simple nature, our method is applicable to pedigrees with complex structure, in which classical parametric linkage methods have difficulty in handling inheritance vectors. While exome-sequencing has demonstrated success in pedigrees with typical Mendelian inheritance and complete penetrance, additional approaches, such as we describe, are required for more complex patterns of disease segregation [8].

There are important limitations of our study. First, our search for rare mutations was performed in a single pedigree and not validated in other pedigrees. While we identified independent *PLB1* alleles associated with risk of RA in an outbred European population using a large-scaled GWAS meta-analysis, it would not directly support the risk of *PLB1* p.G755R mutation on RA. As additional families with familial forms of RA (or related conditions) are identified, it will be important to apply similar unbiased approaches to search for mutations in *PLB1* or *PLB1*-like genes. Second, no single genetic variant achieved a genome-wide level of significance in the GWAS meta-analysis and targeted sequencing in outbred populations. However, whether the same conservative significance thresholds should be applied to our study design is a matter of debate. While we found genetic evidence across the three stages of our study, future genetic studies are required to confirm that *PLB1* alleles definitively contribute to risk of RA. Especially, considering the recent studies reporting that large sample size would be necessary for rare variants analysis in the complex diseases [46], additional accumulation of the subjects in *PLB1* target exon sequencing would be desirable. Finally, we did not perform any functional studies of the variants we identified. Future functional studies will be important to determine if these are gain-of-function or loss-of-function alleles.

In conclusion, our study demonstrates significant contributions of rare, low-frequency, and common alleles of *PLB1* to risk of RA by coordinately assessing a consanguineous pedigree with RA and outbred RA cases-control cohorts. We also introduced novel statistical methodologies to assess rare variants in complex pedigrees with uncertain patterns of inheritance. Our study should contribute to our understanding of the causal variants in the pathogenesis of complex diseases.

## Materials and Methods

### Ethics statement

Our study was approved by the Institutional Review Board of Brigham & Women's Hospital and Tishreen Hospital. All the enrolled subjects provided written informed consent for the participation of the study. For the patients from Syria, written informed consent was provided in Arabic and the study was approved by the Syrian Ministry of Health. Blood samples were collected according to protocols approved by local institutional review boards.

### Samples

We report a 4-generation, consanguineous pedigree in which 8 of 49 individuals are affected with RA (Figure 2 and Table S1). The pedigree members were recruited by a board-certified rheumatologist from Tishreen Hospital, Damascus, Syria. All RA cases fulfilled the revised criteria of the American Rheumatism Association for RA [47]. ACPA titer was determined by by direct assay using QUANTA Lite™ CCP3 IgG ELISA (INOVA Diagnostics, San Diego, CA). In this study, we enrolled 24 living pedigree members including 5 affected cases (II:12, III:3, III:17, IV:5, IV:9), 1 ACPA-positive unaffected subject (III:2), and 18 ACPA-negative unaffected subjects (II:5, II:6, III:1, III:4, III:5, III:6, III:9, III:11, III:12, III:13, III:14, III:19, III:20, IV:1, IV:2, IV:3, IV:4, IV:7; Figure 2).

For the case-control association analysis of RA in the European populations, we studied 8,875 RA cases and 29,367 matched controls for GWAS meta-analysis (Table S4). Six GWAS data has been previously published [2]; additional five GWAS datasets are previously unpublished [29], [32], [33], as described in more detail below. The 1,088 RA cases and 1,088 matched controls for exon sequencing of *PLB1* represent a subset of patients with GWAS data (Table S4). All the subjects were confirmed to be of European origins using both self-reported ethnicities and the results of principal component analysis (PCA). Part of the subjects in the GWAS and exon sequencing were included in previous studies with detailed descriptions of the cohorts [2], [4], [7], [29], [32], [33]. There was an overlap of the RA cases involved in the GWAS meta-analysis and exon sequencing (*n* = 342).

### Whole-genome SNP genotyping and quality control of the consanguineous pedigree with RA

Whole-genome SNP genotyping was conducted for all the available 24 pedigree members using Illumina HumanOmniExpress Genotyping BeadChip (Illumina, San Diego, CA; Figure 1A). We applied call-rate cutoff thresholds of ≥0.98 for samples and 0.99≥ for SNPs, and filtered out subjects with excess heterozygosity. We used GWAS data to confirm the relationships among all pedigree members using “–genome” option implemented in PLINK v1.07. We excluded monomorphic SNPs in the genotyped pedigree members.

### IBD mapping of the consanguineous pedigree with RA

To find the region harboring the ancestral haplotype that co-segregates with affected cases, we conducted IBD mapping of the consanguineous pedigree using whole-genome SNP genotyping data (Figure 1A). After applying LD pruning of the SNPs with *r^2^*≥0.9 using LD information obtained from HapMap Phase II CEU subjects, we applied homozygosity mapping of the affected RA cases and the ACPA-positive unaffected subject using “–homozyg” option implemented in PLINK v1.07, a classical non-parametric linkage analysis assuming a recessive mode of disease inheritance [48].

We developed a novel IBD mapping method which extends homozygosity mapping to include any type of inheritance mode. This method, which is based on the “SNP streak” approach to assess homogeneity of the adjacent SNP genotypes on the ancestral haplotype [22], [23], is applicable without prior estimation of inheritance mode and mutation penetrance. Our method uses genome-wide SNP data to search the regional IBD stretches that lose one or both homozygous genotypes in affected cases using a sliding window approach (Figure 3A). The window spanning 1 Mbp bin was defined as IBD when all the SNP genotypes in this bin followed the rule mentioned above with exception of no more than 1 SNP, and the IBD stretch was defined when the IBD window continued beyond ≥2 Mbp length.

We further extended the method to utilize genotype data from unaffected pedigree members as well as affected cases. Within the identified IBD stretches shared among affected cases, our method is able to impute presence or absence of the ancestral haplotype in the other unaffected subjects, by checking whether IBD stretch remains or not after inclusion of each of the unaffected subjects separately. We assessed significance of the probability to observe the IBD stretch shared among 5 ACPA-positive RA cases and 1 ACPA-positive unaffected subject while only 6 of 16 ACPA-negative unaffected subjects by a permutation procedure (×10,000 iterations). For each of the iteration steps, we randomly selected 6 members from the pedigree as “affected” subjects, and assessed whether at least one of the IBD stretches observed among these 6 “affected” subjects were observed in ≤6/18 of the other “unaffected” subjects. Java™ software for this novel IBD mapping method is available at http://plaza.umin.ac.jp/~yokada/datasource/software.htm.

We did not apply parametric linkage analysis methods for SNP genotype data such as Merlin [49], since the software did not work properly due to the complex pedigree structure including multiple loops.

### Whole-exome sequencing of the RA cases in the consanguineous pedigree

To search for the causal risk mutation in the pedigree, we performed whole-exome sequencing for 4 affected RA cases in the pedigree (II:12, III:3, III:17, IV:9; Figure 1B). DNA library preparation and target exome capture were conducted using the Agilent SureSelect All Exon kit v2 (Agilent Technologies, Santa Clara, CA), which covers 44.9 Mbp of human exon regions. Sequencing was run on Illumina HiSeq2000 (Illumina, San Diego, CA) at the Broad Institute of MIT and Harvard (Cambridge, MA). Sequencing reads were aligned to the Human Reference Genome (UCSC hg19) using Burrows-Wheeler Aligner (BWA) algorithm [50]. Sequence read filtering and variant calling was done using the GATK pipeline as described elsewhere [51], [52], and snpEff was used for variant annotation [53]. Calling of CNV was conducted by using the ExomeDepth software version 0.9.7 [54]. Whole-exome sequencing data of the pedigree is available to other researchers upon request.

Filtering of the identified variants was conducted according to the following processes: (i) variants likely to be pathogenic (missense, nonsense, frameshift Indels, or splice-site acceptor/donor); (ii) variants not registered in the databases (dbSNP v132, 1000 Genomes Project Phase I data [24], and ESP5400 [25]) with non-reference allele frequency ≥0.01; (iii) for Indels, ones not located ±5 bp of known variants; and (iv) variants of which ≥1 non-reference alleles were observed in all the exome sequenced 4 RA cases.

### Validation iPLEX*™* assay of the exome-driven variants

To efficiently validate the results of whole-exome sequencing, we selected the 3 candidate causal variants that were included in the IBD stretches defined using SNP genotype data from 5 ACPA-positive RA cases and 1 ACPA-positive unaffected subject (Figure 1C). We conducted iPLEX™ validation assay (Montréal, Canada) for these selected variants using the available 22 pedigree members except for the 2 ACPA-negative unaffected subjects (IV:3 and IV:4), due to genomic DNA degradation. Relative risk of each validated variant was evaluated using mid-P value of Fisher's exact test, which has more unbiased type I error and higher statistical power compared to original Fisher's exact test [55]. Java™ software for mid-P value of Fisher's exact test is available at http://plaza.umin.ac.jp/~yokada/datasource/software.htm.

### Association analysis of the *PLB1* locus using European RA GWAS meta-analysis

To evaluate RA genetic risk of the *PLB1* locus in the general populations, we referred to the results of the currently conducted RA GWAS meta-analysis of the European populations enrolling 8,875 RA cases and 29,367 controls from 11 studies (Figure 1D and Table S4). Five new unpublished GWAS datasets (*n* = 3,427 cases and 6,837 controls) from ReAct [29], Dutch (including AMC, BeSt, LUMC, and DREAM) [29], anti-TNF response to therapy collection (ACR-REF: BRAGGSS, BRAGGSS2, ERA, KI, and TEAR) [29]-[31], the Consortium of Rheumatology Researchers of North America (CORRONA) [32], and Vanderbilt RA case-control cohorts [33] were included along with 6 previously published GWAS datasets (*n* = 5,448 cases and 22,530 controls) [2], [29]. All GWAS data was filtered using the same criteria as described elsewhere [2]–[4], including sample and SNP call-rate cutoffs, exclusion of closely-related or outlier subjects, and MAF and Hardy-Weinberg equilibrium cutoffs for SNPs.

After applying QC criteria to each GWAS, whole-genome genotype imputation was performed by minimac [56] using 1000 Genome Project Phase I (α) European data as a reference [24]. We excluded imputed SNPs with MAF<0.005 or imputation score of *Rsq*<0.5 from each GWAS. Associations of the SNPs with RA were evaluated by logistic regression models assuming additive effects of the allele dosages, including top 5 principal components as covariates using mach2dat v1.0.16 (see URL). Meta-analysis was performed for the SNPs available in ≥50% of the studies, by an inverse-variance method assuming a fixed-effects model on the effect sizes of the alleles dosages using the Java™ source code implemented by the authors [57]. Double genomic control (GC) correction was carried out using the inflation factor (λ_GC_) obtained from the results of each GWAS and the GWAS meta-analysis.

The regional significance threshold was determined by a permutation procedure (permutation *P* = 0.05 with ×10,000 iterations). Case-control phenotype labels were shuffled for each GWAS dataset separately, and the distribution of the smallest *P*-values of the SNPs from respective iteration steps was evaluated. Conditional analysis was conducted by consecutively including the allele dosages of the top-associated SNPs in the *PLB1* locus as covariates in a forward-type stepwise logistic regression approach until no significant regional association was observed after conditioning (α = 0.01). Haplotype analysis was conducted by incorporating estimated haplotype dosages consisting of the two non-coding SNPs in *PLB1* (rs116018341 and rs116541814) as independent variables, as described elsewhere [58].

We obtained chromatin immunoprecipitation followed by sequencing (ChIP-seq) assay peaks of H3K4me3 from NIH Roadmap Epigenomics Mapping Consortium [59], and assessed overlap of the SNPs in the *PLB1* locus with H3K4me3 peaks in primary T_reg_ cells, as described elsewhere [60]. Peak overlap enrichment of the SNPs (and SNPs in absolute LD with it; *r^2^* = 1.00) were compared to the neighboring SNPs (±2 Mbp). We physically slid H3K4me3 peak positions by 100 bp bins within ±2 Mbp regions of the SNPs, and assessed overlap with H3K4me3 peaks for each sliding step. Significance of overlap in the original peak positions was evaluated by one-sided exact test assuming enrichment of overlap.

### Deep exon sequencing of *PLB1* in European RA case-control cohort

To evaluate contribution of *PLB1* protein-coding variants on the risk of general RA cases, we conducted deep exon sequencing of *PLB1* using genetically matched 1,088 RA cases and 1,088 controls from the European populations (Figure 1E and Table S4). These subjects were collected as a part of the PGRN sequencing project, as described elsewhere [Diogo D. et al. Manuscript in preparation]. All subjects were determined as European ancestry based on PCA conducted along with HapMap Phase III samples as reference populations. RA cases and controls were matched based on the Euclidean distances in all case-control pairs along 10 eigenvalue-weighted PCs. DNA library preparation and target exon capture was conducted using NimbleGen Sequence Capture technology (Roche NimbleGen, Madison, WI), along with another ∼850 genes related to autoimmune diseases as a part of the PGRN sequencing project. Sequencing was run on Illumina HiSeq2000 (Illumina, San Diego, CA) at the Genome Institute at Washington University in St. Louis. Sequencing reads were aligned to the Human Reference Genome (UCSC hg19) using the BWA algorithm [50], and duplicated reads were excluded using Picard (see URL). Sequence read filtering and variant calling was done using SAMtools v1.16 and VarScan v2.2.9 [61], [62]. Variants were annotated based on *PLB1* transcript (NM_153021) using ANNOVAR [63]. We selected rare protein-coding variants of *PLB1* (MAF<0.01) and evaluated gene-based association signal on RA risk by sets of widely-used rare variants tests, including the burden test, variable threshold test [36], frequency-weighted test [37], C-alpha test [38], and SKAT [39], using PLINKQ-SEQ (with ×100,000 iterations) and SKAT [39] software. PLB1 protein domains were obtained from the Pfam protein families database for UniProt entry Q6P1J6 [41]. Deep exon sequencing data of *PLB1* is available to other researchers upon request.

### Web resources

The URLs for data presented herein are as follows:

Java™ software for the IBD mapping and mid-P value of Fisher's exact test, http://plaza.umin.ac.jp/~yokada/datasource/software.htm

Online Mendelian Inheritance in Man (OMIM), http://omim.org/

PLINK, http://pngu.mgh.harvard.edu/~purcell/plink/

GATK, http://www.broadinstitute.org/gatk/

dbSNP, http://www.ncbi.nlm.nih.gov/snp/

1000 Genomes Project, http://www.1000genomes.org/

NHLBI Grand Opportunity Exome Sequencing Project, https://esp.gs.washington.edu/drupal/

minimac, http://genome.sph.umich.edu/wiki/Minimac

mach2dat, http://www.sph.umich.edu/csg/abecasis/MACH/index.html

NIH Roadmap Epigenomics Mapping Consortium, http://www.roadmapepigenomics.org/

Picard, http://picard.sourceforge.net/index.shtml

Annovar, http://www.openbioinformatics.org/annovar/

ExomeDepth, http://cran.r-project.org/web/packages/ExomeDepth/index.html

PLINK-SEQ, http://atgu.mgh.harvard.edu/plinkseq/

SKAT, http://www.hsph.harvard.edu/skat/

Pfam protein families database, http://pfam.sanger.ac.uk/

eQTL Browser, http://eqtl.uchicago.edu/Home.html

Blood eQTL Browser, http://genenetwork.nl/bloodeqtlbrowser/

## Supporting Information

Figure S1 **Protein structure of** ***PLB1*** **and RA risk variant.** *PLB1* protein has three GDSL-like lipase domains which have essential biological roles in lysophopholipase activity of the protein. The second GDSL-like lipase domain included p.G755R mutation identified in the consanguineous RA pedigree (the black triangle).
(TIF)

Table S1 **Characteristics of RA cases and a ACPA-positive unaffected subject in the consanguineous pedigree with RA.**
(DOCX)

Table S2 **Results of IBD mapping for the consanguineous pedigree with RA.**
(DOCX)

Table S3 **A list of the filtered variants from whole exome sequencing.**
(DOCX)

Table S4 **Characteristics of the subjects in European RA case-control cohorts.**
(DOCX)

Table S5 **Rare variants obtained from deep exon sequencing of** ***PLB1*** **in the European RA case-control cohort.**
(DOCX)

Checklist S1 **PRISMA checklist.**
(DOC)
