# Supplementary figures and images for: Integration of Sequence Data from a Consanguineous Family with Genetic Data from an Outbred Population Identifies PLB1 as a Candidate Rheumatoid Arthritis Risk Gene
Source: PLoS One. 2014 Feb 10;9(2):e87645. doi: 10.1371/journal.pone.0087645 (PMC3919745; doi:10.1371/journal.pone.0087645)

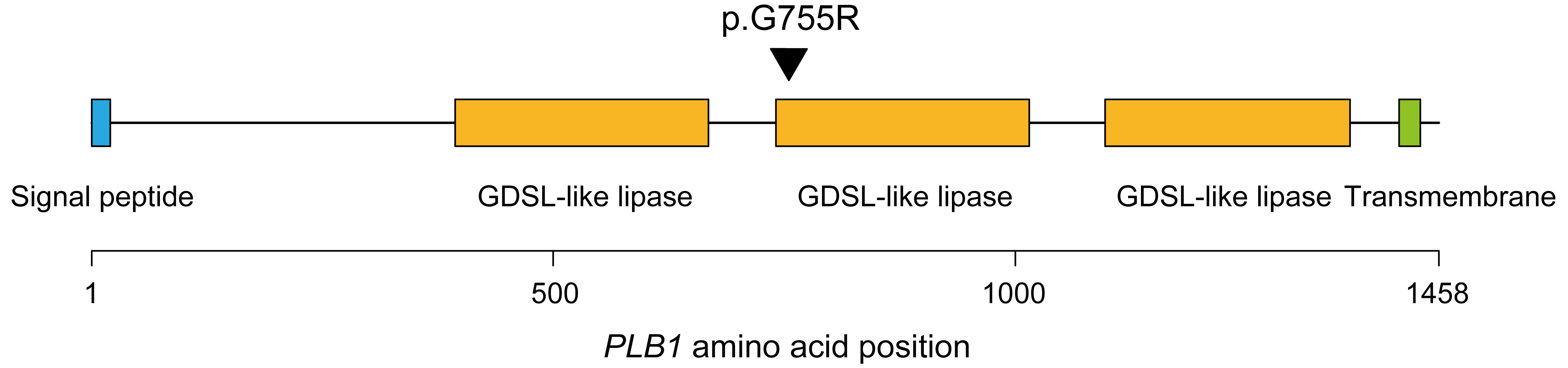

Supplement: Figure S1 — Protein structure of PLB1 and RA risk variant. PLB1 protein has three GDSL-like lipase domains which have essential biological roles in lysophopholipase activity of the protein. The second GDSL-like lipase domain included p.G755R mutation identified in the consanguineous RA pedigree (the black triangle). (TIF) [file pone.0087645.s001.tif]
