# Supplementary material for: Integration of Sequence Data from a Consanguineous Family with Genetic Data from an Outbred Population Identifies PLB1 as a Candidate Rheumatoid Arthritis Risk Gene
Source: PLoS One. 2014 Feb 10;9(2):e87645. doi: 10.1371/journal.pone.0087645 (PMC3919745; doi:10.1371/journal.pone.0087645)
Supplement: Table S1 — Characteristics of RA cases and a ACPA-positive unaffected subject in the consanguineous pedigree with RA. (DOCX) [file pone.0087645.s002.docx]

**Table S1.** Characteristics of RA cases and a ACPA-positive control in the consanguineous pedigree with RA.

| Subject | II:12 | III:2 | III:3 | III:17 | IV:5 | IV:9 |
| --- | --- | --- | --- | --- | --- | --- |
| Disease status | RA | ACPA+ control | RA | RA | RA | RA |
| Age at sampling (year) | 72 | 29 | 33 | 50 | 26 | 34 |
| Age at RA onset (year) | 60 | - | 31 | 38 | 18 | 30 |
| Gender | female | female | female | female | male | female |
| *PLB1* mutation | G755R/WT | G755R/WT | G755R/WT | G755R/WT | G755R/WT | G755R/WT |
| ACPA titer (units)^a^ | 95.4 | 83.3 | 110.6 | 107.7 | ++ | 26.4 |

^a^ ACPA titer ≥20, ≥40, ≥60 units are assigned as "weak positive", "moderate positive", "strong positive", respectively. ACPA titer too high to be measured is represented as "++".

RA; rheumatoid arthritis, ACPA; anti-citrullinated protein antibodies.
