# Supplementary material for: Integration of Sequence Data from a Consanguineous Family with Genetic Data from an Outbred Population Identifies PLB1 as a Candidate Rheumatoid Arthritis Risk Gene
Source: PLoS One. 2014 Feb 10;9(2):e87645. doi: 10.1371/journal.pone.0087645 (PMC3919745; doi:10.1371/journal.pone.0087645)
Supplement: Table S2 — Results of IBD mapping for the consanguineous pedigree with RA. (DOCX) [file pone.0087645.s003.docx]

**Table S2.** Results of IBD mapping for the consanguineous pedigree with RA.

| Subjects observed for IBD stretches | No. IBD stretches | Total length of IBD stretches (Mbp) | Proportion to human genome |
| --- | --- | --- | --- |
| 5 RA case and 1 ACPA+ control | 14 | 115.9 | 3.7% |
| 5 RA case and 1 ACPA+ control and ≤ 15/18 ACPA- controls | 13 | 114.4 | 3.7% |
| 5 RA case and 1 ACPA+ control and ≤ 12/18 ACPA- controls | 9 | 92.9 | 3.0% |
| 5 RA case and 1 ACPA+ control and ≤ 9/18 ACPA- controls | 2 | 11.0 | 0.35% |
| 5 RA case and 1 ACPA+ control and ≤ 6/18 ACPA- controls | 1 | 2.4 | 0.08% |
| 5 RA case and 1 ACPA+ control and ≤ 3/18 ACPA- controls | 0 | 0 | 0% |
| 5 RA case and 1 ACPA+ control and ≤ 0/18 ACPA- controls | 0 | 0 | 0% |

RA; rheumatoid arthritis, ACPA; anti-citrullinated protein antibodies.
