# Supplementary material for: Integration of Sequence Data from a Consanguineous Family with Genetic Data from an Outbred Population Identifies PLB1 as a Candidate Rheumatoid Arthritis Risk Gene
Source: PLoS One. 2014 Feb 10;9(2):e87645. doi: 10.1371/journal.pone.0087645 (PMC3919745; doi:10.1371/journal.pone.0087645)
Supplement: Table S3 — A list of the filtered variants from whole exome sequencing. (DOCX) [file pone.0087645.s004.docx]

**Table S3.** A list of the filtered variants from whole exome sequencing.

| Gene^a^ | Chr | Position (bp)^b^ | Allele | Amino acid change |
| --- | --- | --- | --- | --- |
|  |  |  | Ref/Alt |  |
| *PDE4DIP* | 1 | 145,075,775 | G/A | P30S |
| *CD1E* | 1 | 158,326,666 | C/T | R127C |
| *PAPPA2* | 1 | 176,640,191 | C/T | R693W |
| *RGS18* | 1 | 192,129,537 | G/A | G84D |
| *PLB1* | 2 | 28,816,563 | G/C | G755R |
| *NIM1* | 5 | 43,245,938 | C/T | R21W |
| *FGF10* | 5 | 44,388,667 | G/C | L40V |
| *KIAA1549* | 7 | 138,603,473 | G/A | P250L |
| *FGFBP3* | 10 | 93,668,630 | C/T | A33T |
| *LCOR* | 10 | 98,715,020 | C/G | L215V |
| *TRABD* | 22 | 50,633,411 | C/T | T121M |
| *MAPK11* | 22 | 50,708,703 | C/T | G7S |
| *AMOT* | X | 112,022,297 | C/CAGG | P1028PL |
| *ANKRD58* | X | 118,893,513 | G/A | G295S |

^a^ Genes of which mutations were shared among 4 RA cases and also included in the IBD stretches of these 4 RA cases were indicated.

^b^ Based on NCBI Build 37/hg19.

RA; rheumatoid arthritis, ACPA; anti-citrullinated protein antibodies.
