# Supplementary material for: Integration of Sequence Data from a Consanguineous Family with Genetic Data from an Outbred Population Identifies PLB1 as a Candidate Rheumatoid Arthritis Risk Gene
Source: PLoS One. 2014 Feb 10;9(2):e87645. doi: 10.1371/journal.pone.0087645 (PMC3919745; doi:10.1371/journal.pone.0087645)
Supplement: Table S4 — Characteristics of the subjects in European RA case-control cohorts. (DOCX) [file pone.0087645.s005.docx]

**Table S4.** Characteristics of the subjects in European RA case-control cohorts.

| Study | Cohort | No. RA cases | No. Controls | Total | λ_GC_ in GWAS^a^ | Reference |
| --- | --- | --- | --- | --- | --- | --- |
| GWAS meta-analysis | Brass | 483 | 1,631 | 2,114 | 1.015 | Stahl et al. Chen et al. |
|  | Canada | 589 | 1,554 | 2,143 | 1.002 | Stahl et al. Chen et al. |
|  | Eira | 1,097 | 1,044 | 2,141 | 0.991 | Stahl et al. Chen et al. |
|  | Narac1 | 863 | 1,191 | 2,054 | 1.017 | Stahl et al. Chen et al. |
|  | Narac2 | 896 | 6,603 | 7,499 | 1.023 | Stahl et al. Chen et al. |
|  | WTCCC | 1,520 | 10,507 | 12,027 | 1.043 | Stahl et al. Chen et al. |
|  | ReAct | 275 | 804 | 1,079 | 0.992 | Cui et al. |
|  | AMC, BeSt, LUMC, DREAM | 1,172 | 1,684 | 2,856 | 1.023 | Cui et al. |
|  | BRAGGSS, BRAGGSS2, ERA, KI, TEAR | 347 | 264 | 611 | 1.026 | Cui et al. Bathon et al. Moreland et al. |
|  | Corrona | 894 | 1,838 | 2,732 | 1.001 | Fisher et al. |
|  | Vanderbilt | 739 | 2,247 | 2,986 | 0.987 | Carroll et al. |
|  | Total | 8,875 | 29,367 | 38,242 | 1.034 | - |
| *PLB1* exon sequencing | PGRN | 1,088 | 1,088 | 2,176 | - | - |

^a^ Used for double genomic control (GC) correction of GWAS and meta-analysis results.

RA; rheumatoid arthritis, GWAS; genome-wide association study.
