# Supplementary material for: Integration of Sequence Data from a Consanguineous Family with Genetic Data from an Outbred Population Identifies PLB1 as a Candidate Rheumatoid Arthritis Risk Gene
Source: PLoS One. 2014 Feb 10;9(2):e87645. doi: 10.1371/journal.pone.0087645 (PMC3919745; doi:10.1371/journal.pone.0087645)
Supplement: Table S5 — Rare variants obtained from deep exon sequencing of PLB1 in the European RA case-control cohort. (DOCX) [file pone.0087645.s006.docx]

**Table S5.** Rare variants obtained from deep exon sequencing of PLB1 in the European RA case-control cohort.

^a^ Based on NM_153021.

^b^ Most damaging predictions are indicated.

Chr; chromosome, MAC; minor allele count, MAF; minor allele frequency, Ref; reference, Alt; alternative.
